# Neurosurgical Outcomes of Epilepsy Surgery Between 2017 and 2025: A Retrospective ESSQ-19 Study From a Tertiary Care Center

**DOI:** 10.7759/cureus.116081

**Published:** 2026-09-10

**Authors:** Kiran Kumari, Govind Mangal, Gaurav Gupta, Isha Purohit

**Affiliations:** 1 Neurosurgery, Geetanjali Medical College and Hospital, Udaipur, IND

**Keywords:** adult epilepsy surgery, drug-resistant epilepsy, epilepsy research, medication-refractory epilepsy, neurosurgical outcome

## Abstract

Introduction

Epilepsy is a common neurological disorder. Despite being on anti-epileptic drug therapy, a subset of patients continue to experience seizures. In such patients with drug-resistant epilepsy, surgery remains a viable treatment option. Patient-reported satisfaction provides an important perspective on post-epilepsy surgery outcomes. This study aimed to descriptively assess postoperative patient satisfaction using the validated Epilepsy Surgery Satisfaction Questionnaire-19 (ESSQ-19).

Materials and methods

This was a retrospective observational study conducted at a tertiary care centre in India. This study enrolled a total of 45 patients who underwent epilepsy surgery between 2017 and 2025. Details such as clinical and demographic information were obtained from the medical records. Patient satisfaction was assessed through telephone interviews using the ESSQ-19 questionnaire. Descriptive analyses were performed to evaluate the questionnaire responses.

Result

In the subset of patients who underwent epilepsy surgery between 2017 and 2025 (27 males, 60%, and 18 females, 40%), the mean age at the time of questionnaire administration was 30.73 ± 9.40 years. A major subset of patients had undergone temporal lobe surgery (n=35, 77.8%), and at the time of questionnaire administration were not on any anti-epileptic drugs (n=25, 55.5%). The Epilepsy Surgery Satisfaction Questionnaire-19 (ESSQ-19) demonstrated favourable patient satisfaction (Summary Score 84.1±21.7). Out of the four domains assessed, a high mean score was reported in the surgical complication domain (89.2±21.1) and the seizure control domain (85.0±27.6). Additionally, comparatively lower scores were observed in the psychosocial domain (79.9±24.1). Most patients expressed being extremely satisfied (N=15, 33.3%), or very satisfied (N=13, 28.9%) with their surgery.

Conclusion

In this single-centre retrospective study, patients reported favourable postoperative satisfaction following epilepsy surgery, with favourable mean scores observed in the surgical complication and seizure control domains. The Epilepsy Surgery Satisfaction Questionnaire-19 (ESSQ-19) may provide a useful patient-centred tool for assessing patient satisfaction following epilepsy surgery.

## Introduction

With an estimated prevalence of 50 million, epilepsy remains one of the most common neurological disorders globally [1]. Studies have estimated that with the use of anti-epileptic drugs (AEDs), approximately 60% to 70% of patients suffering from epilepsy respond, while 30% to 40% become resistant to medications [2-4]. According to the International League Against Epilepsy (ILAE), drug-resistant epilepsy is defined as failure of adequate trials of two tolerated and appropriately chosen and used AED schedules (whether as monotherapies or in combination) to achieve sustained seizure freedom [5]. Individuals suffering from drug-resistant epilepsy have increased tendencies to suffer from psychiatric and mood disorders, along with cognitive impairment [6]. These individuals also suffer from a higher burden of morbidity and mortality [7]. Such factors can significantly impair the quality of life in patients suffering from drug-resistant epilepsy.

In such patients, surgical treatment may provide seizure reduction or seizure freedom [6,8]. Although seizure control is an important outcome of epilepsy surgery, it does not capture the patients’ satisfaction with the treatment [9]. While quality of life measures assess the clinical outcome of treatment in terms of symptomatic and functional well-being, treatment satisfaction is a patient-reported outcome that reflects a patient’s own perception of the treatment process and its outcomes [10,11]. Prior assessment of satisfaction with epilepsy surgery has largely been measured with nonvalidated and heterogeneous measures, highlighting the need for a surgery-specific instrument [9,10].

The Epilepsy Surgery Satisfaction Questionnaire-19 (ESSQ-19) specifically addresses these limitations [12]. It is a multidimensional assessment tool that covers several aspects of epilepsy surgery in the form of four domains: Seizure control, Psychosocial functioning, Surgical complications, and Recovery from surgery. Since its development, the ESSQ-19 questionnaire has been validated across multiple countries and languages to assess satisfaction with epilepsy surgery [12-16]. However, despite the high burden of drug-resistant epilepsy in countries such as India, patient satisfaction after epilepsy surgery remains underreported [17]. The primary objective of this study was to descriptively assess patient satisfaction after epilepsy surgery across all four domains of the Epilepsy Surgery Satisfaction Questionnaire (ESSQ-19), which includes the seizure control, psychosocial functioning, surgical complication, and recovery from surgery domains, along with a global satisfaction item. The secondary objective of this study was to describe the demographic, clinical, and surgical characteristics in a subset of patients who underwent epilepsy surgery at a tertiary care centre in India.

## Materials and methods

Study setting

This was a retrospective observational study conducted in the Department of Neurosurgery at Geetanjali Medical College and Hospital. The study began after obtaining approval from the institute’s Human Research Ethics Committee (HREC). The study was conducted in accordance with the Declaration of Helsinki (Approval No. GU/HREC/EC/2025/2732); it enrolled all patients who underwent epilepsy surgery from 2017 to 2025.

Patient selection and eligibility criteria

The study began after receiving approval from the Institute’s Ethical Committee. This study included 45 patients who underwent epilepsy surgery between January 2017 and February 2025 with a minimum postoperative follow-up of more than one year. Patient information, including clinical and demographic details, was obtained from the Medical Records Department while maintaining strict confidentiality to safeguard patient information. Informed consent was obtained before enrolment of the patients in the study. After applying the eligibility criteria, a total of 45 patients were enrolled. The details regarding the eligibility criteria are presented in Table 1.

**Table 1:** Inclusion and exclusion criteria for participant selection ESSQ-19: Epilepsy Surgery Satisfaction Questionnaire-19

Inclusion Criteria	Exclusion Criteria
Individuals who underwent surgery for drug-resistant epilepsy at the tertiary care centre between January 2017 and February 2025	Individuals who refused to provide informed consent
Age ≥ 18 at the time of questionnaire administration	Individuals lost to follow-up
Minimum postoperative follow-up of more than 1 year at the time of questionnaire administration	Individuals with incomplete medical records
Ability to understand the ESSQ-19 questionnaire in English language	

Presurgical evaluation

All patients underwent presurgical evaluation at the Department of Neurosurgery and Neurology. The presurgical neurological workup comprised video EEG monitoring and magnetic resonance imaging using an epilepsy protocol. The mean duration of epilepsy before surgery was 8.21 ± 3.64 years.

Epilepsy Surgery Satisfaction Questionnaire (ESSQ-19)

In order to evaluate patient satisfaction, the ESSQ-19 questionnaire developed by Wiebe et al. was used [10]. ESSQ-19 is a patient-reported outcome measure tool that was validated in 229 adults who underwent epilepsy surgery at three Canadian centers and one Swedish center. The four domain scores and the summary score demonstrated internal consistency (Cronbach's α = 0.84-0.95) with good to excellent test-retest reliability (ICC = 0.71-0.85). Additionally, the construct validity was supported by correlation with established quality-of-life measures. The ESSQ-19 questionnaire measures overall patient satisfaction with epilepsy surgery using 19 items, which are divided into four domains and one global item measuring overall satisfaction with the epilepsy surgery. The seizure control domain contains five items addressing seizure control, prevention of accidents or injuries caused by seizures, how the patient feels after a seizure, worry about having a seizure, and whether surgery met the patient's expectations. The psychosocial functioning domain comprises seven items covering social life, performance at work (including domestic work), school or volunteering, self-confidence, cognitive function, mood, overall physical health, and overall quality of life. The surgical complications domain has three items addressing poor balance or coordination, muscle weakness, and decreased sensation (e.g., numbness). The recovery from surgery domain comprises three items addressing speed of recovery in hospital, recovery after discharge from hospital (not including rehabilitation), and rehabilitation after discharge from hospital. The nineteenth item is a global satisfaction item measuring overall satisfaction with the epilepsy surgery. To minimise recall bias of distant postoperative events across varied follow-up time periods, the ESSQ-19 questionnaire asks the patient to respond based on how they’ve felt in the past four weeks or currently. Seizure freedom at the time of study was described as the absence of seizure during the 12 months preceding the questionnaire administration.

Telephone interview

This study began after obtaining ethical approval from the institute’s ethical committee. It recruited patients who underwent epilepsy surgery at our tertiary care centre within the designated study period. After application of the eligibility criteria, a total of 45 patients were scheduled for a telephone interview, which was conducted by a researcher who was blinded to the surgical outcome and clinical data and was not involved in patient care. To avoid any confounding and interference, it was ensured that the questionnaire was conducted in a single interview, and that the primary surgeon and the entire surgical team involved in patient care were not present for the duration of the interview. The ESSQ-19 questionnaire was then administered, and the patient’s responses were recorded verbatim and entered into the database by a researcher who was not a part of the original surgical team in order to ensure blinding. The mean duration of the interview was 34.8 ± 5.5 minutes. The study flow diagram according to STROBE guidelines is presented in Figure 1.

**Figure 1:**
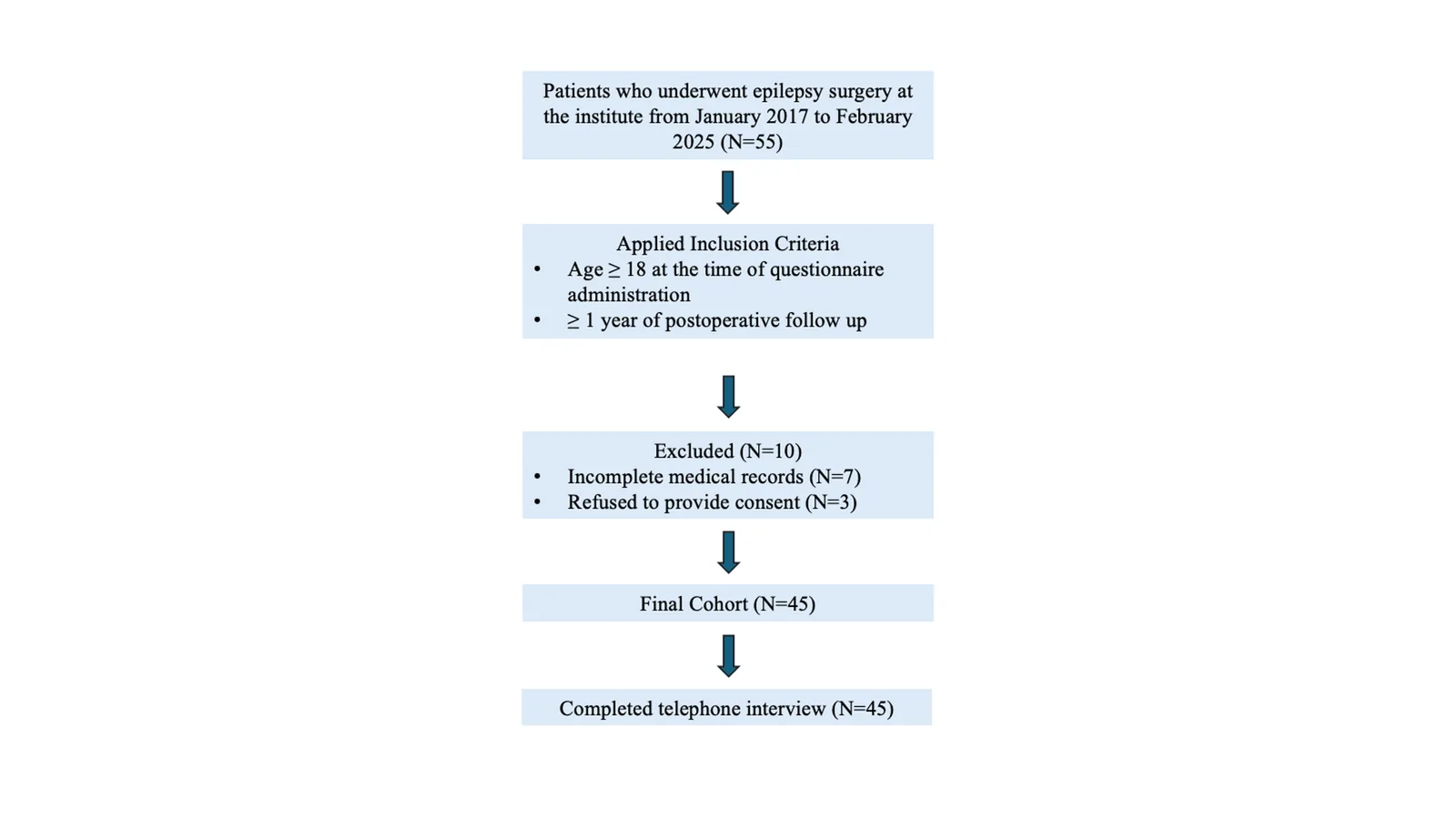
STROBE flow diagram of patient selection STROBE: Strengthening the Reporting of Observational Studies in Epidemiology

Scoring

The Epilepsy Surgery Satisfaction Questionnaire consists of 19 items that measure four domains: Seizure Control, Psychosocial Functioning, Surgical Complications, and Recovery From Surgery. Additionally, it includes a global satisfaction scale that assesses a patient’s overall satisfaction with epilepsy surgery. The responses are graded on a Likert scale, ranging from 1 to 6, 7, or 8 depending on the domain. All responses are converted into values ranging from 0 to 100, where 0 indicates the worst possible satisfaction, and 100 indicates the highest satisfaction. Domains were calculated by averaging the items within each domain, and summary scores were calculated as the mean of the domains. The scoring criteria for the questionnaire are given in Table 2.

**Table 2:** ESSQ-19 scoring system and conversion of questionnaire responses to standardized 0–100 scores ESSQ-19: Epilepsy Surgery Satisfaction Questionnaire-19

Questionnaire Items	Scale	Conversion
Items 1–11, 15–16, 18–19	1–7 Likert scale	1 = 0, 7 = 100
Items 12–14 (surgical complications)	1–6 Likert scale	1 = 0, 6 = 100
Item 17 (rehabilitation)	1–8 scale	1 = 0, 8 = 100

Statistical analysis

The responses were collected and analysed using Microsoft Excel for Mac, version 16.109.2 (Microsoft Corporation, Redmond, WA, USA; Office Home 2024 license). Descriptive statistics were calculated for demographic details of the patients and for each domain, including seizure control, psychosocial functioning, surgical complications, and recovery from surgery, including mean, standard deviation (SD), median, minimum, and maximum scores. The global satisfaction was tabulated separately. All 45 patients completed the questionnaire in a single interview with no missing item responses. Due to the limited sample size, no inferential statistical testing was performed, and no formal comparison between the subgroups was undertaken.

## Results

Clinical and demographic details

After applying the inclusion criteria, a total of 45 patients who underwent epilepsy surgery and completed the ESSQ-19 questionnaire were included in this study. The mean age of participants at the time of surgery was 26.89 ± 9.64 (range 15 to 55 years), and the mean age at the time of questionnaire administration was 30.73 ± 9.40 years. Out of these, 60% were male (N=27), and 40% were female (N=18). The mean duration of epilepsy in these patients was 8.21 ± 3.64 years, and the majority of the patients had undergone temporal lobe surgery (N=35, 77.8%). At the time of questionnaire administration, a majority of the patients were not on any anti-epileptic drugs (N=25, 55.5%). The total mean postoperative follow-up in years was 3.84 ± 1.89 years. The clinical and demographic details of the patients are presented in Table 3.

**Table 3:** The clinical and demographic details of study participants. SD: Standard Deviation; IQR: Interquartile Range

Clinical and Demographic Details	Total Cohort, N=45
Age at the time of surgery	
Mean±SD (years)	26.89 ± 9.64 years (range 15 to 55 years)
Median (IQR)	23.5 (18 to 34)
Age at the time of questionnaire administration	
Mean±SD (years)	30.73 ± 9.40 years (range 19 to 57 years)
Median (IQR)	29 (22.5 to 38)
Male, n (%)	27 (60%)
Female, n (%)	18 (40%)
Mean duration of epilepsy before surgery (years)	8.21 ± 3.64 years
Mean postoperative follow-up (years)	3.84 ± 1.89 years
Telephone interview	
Mean±SD (minutes)	34.8 ± 5.5 minutes
Range	27 to 49
Temporal Lobe surgery, n (%)	35 (77.8%)
Corpus Callosotomy surgery, n (%)	10 (22.2%)
Seizure-free at the time of study, n (%)	28 (62.2%)
Anti-epileptic drugs use at the time of study	
0	25 (55.5%)
1	15 (33.3%)
≥2	5 (11.1%)

Domain and summary scores

The ESSQ-19 questionnaire assessed four domains: Seizure Control, Psychosocial Functioning, Surgical Complications, and Recovery. Across all these domains, the highest mean score was observed in the Surgical Complications domain (89.2 ± 21.1), indicating patient satisfaction with the surgical complications domain. It was followed by Seizure Control, Recovery, and Psychosocial Functioning. The overall summary score was 84.1 ± 21.7. These findings indicate that the highest patient satisfaction scores were observed in the surgical complications and seizure control domains. The data are presented in Table 4.

**Table 4:** The domain-specific scores of ESSQ-19 questionnaire SD: Standard Deviation; ESSQ-19: Epilepsy Surgery Satisfaction Questionnaire-19

Domain	N	Mean	SD	Median	Min	Max
Seizure Control	45	85.0	27.6	96.7	13.3	100
Psychosocial Functioning	45	79.9	24.1	92.9	28.6	100
Surgical Complications	45	89.2	21.1	93.3	0.0	100
Recovery	45	82.4	23.2	89.7	15.9	100
Summary Score	45	84.1	21.7	93.2	17.5	99.4
Global Satisfaction (Q19)	45	77.41	23.3	83.3	16.7	100

Global satisfaction

The majority of patients reported being extremely satisfied (N=15, 33.3%) with their surgery. The patients' responses to the global satisfaction item (Q19) are reported in Figure 2 and Table 5.

**Figure 2:**
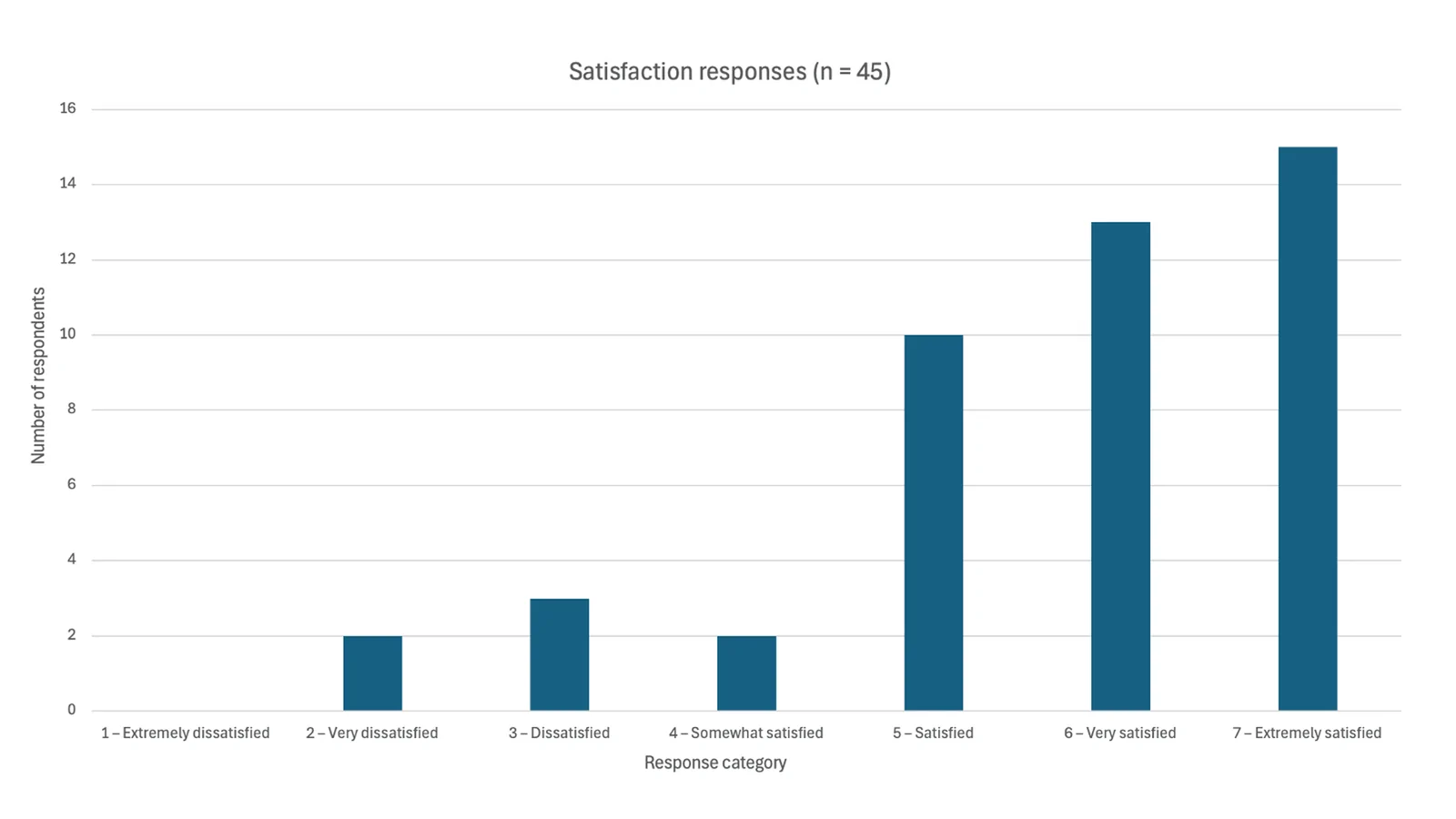
Distribution of responses according to the ESSQ-19 global satisfaction item (Q19), N=45. Responses were graded on a 7-point Likert scale from 1 (extremely dissatisfied) to 7 (extremely satisfied). ESSQ-19: Epilepsy Surgery Satisfaction Questionnaire-19

**Table 5:** Distribution of global satisfaction score

Response category	N	%
1 – Extremely dissatisfied	0	0.0
2 – Very dissatisfied	2	4.4
3 – Dissatisfied	3	6.7
4 – Somewhat satisfied	2	4.4
5 – Satisfied	10	22.2
6 – Very satisfied	13	28.9
7 – Extremely satisfied	15	33.3
Total	45	100

## Discussion

This was a retrospective, single-centre, observational study which was conducted at a tertiary care centre in India. The primary aim of this study was to descriptively assess patient satisfaction following epilepsy surgery using a pre-validated Epilepsy Surgery Satisfaction Questionnaire (ESSQ-19). The study assessed patient satisfaction across four domains: Seizure Control, Psychosocial functioning, Surgical Complication, and Recovery. Overall, our study reported favourable patient satisfaction across all domains, with high mean scores reported in the surgical complication (89.2 ± 21.1) and seizure control domains (85 ± 27.6). The summary score in our study was 84.1 ± 21.7, which was comparable to that reported in the Italian validation cohort (83.7 ± 13.3) and to the Taiwanese cohort (82.5 ± 14.5). This makes our findings consistent with other studies that evaluated patient satisfaction using the ESSQ-19 questionnaire [12,15].

However, the mean score in the psychosocial-functioning domain was comparatively low when compared to other domains (79.9 ± 24.1). In the ESSQ-19 questionnaire, the psychosocial domains contain seven questions pertaining to different aspects of a patient’s life, such as their social life, performance at work, self-confidence, cognitive function, mood, and overall physical health and overall quality of life. This highlights the importance of considering other quality of life outcomes rather than relying on seizure control alone.

In the literature, mood disorders such as depression and anxiety are found to be highly prevalent in individuals with epilepsy, with a substantial proportion persisting even in the postoperative period. In a review done by Rayner and Wilson, it was reported that post-epilepsy surgery, approximately 30% of patients suffered from major depression, with psychosocial recovery lagging behind seizure control [18]. Although the rates of depression declined across the follow-up periods, moderate to severe depression persisted in patients with postoperative seizures. They identified several perioperative risk factors for depression, such as a history of affective disturbances in mood and poor family dynamics. They highlighted the importance of a comprehensive presurgical workup, along with frequent postsurgical follow-ups in order to recognise risk factors and maximise quality of life outcomes.

In another study by Bakhtiar et al., evaluating the postoperative quality of life in patients who underwent temporal lobe epilepsy surgery, an interesting finding emerged [19]. Although patients with seizure freedom were associated with better quality of life outcomes, a negative correlation was found between depression, anxiety, and quality of life outcomes, suggesting that higher levels of depression and anxiety had a greater effect on quality of life than seizure frequency, severity, or chronicity. In another systematic review by Hue et al. comparing depression after temporal lobe surgery, they noted that the incidence of de novo depression after surgery ranged up to 38% across a maximum follow-up period of nine years [20]. The authors also noted significant limitations regarding the sample size, presence of confounding variables, and lack of control groups. Nevertheless, the comparatively low scores in the psychosocial domain, and the above-mentioned studies address the overshadowing of psychosocial outcomes by seizure control outcomes, thus highlighting the importance of providing emotional support, including adequate psychiatric rehabilitation post-surgery. However, while interpreting the results, it should be noted that due to the absence of a preoperative baseline score, comparatively low psychosocial scores should be interpreted as an observational pattern, which warrants future prospective investigations, rather than as evidence of poor postoperative care.

Patient satisfaction post-surgery remains relatively underreported. Our study contributes to the literature by documenting patient satisfaction post-epilepsy surgery using a pre-validated questionnaire, highlighting the importance of patient-reported outcomes.

Limitations

This study has a few limitations which should be considered when interpreting the results. First, this was a retrospective, single-centre study, with a relatively small sample size (N=45). This limits the ability to conduct formal subgroup analysis and for the descriptive findings to be generalized to other patient populations. Second, the lack of a detailed preoperative baseline assessment limits the ability of this study to determine whether the patients’ postoperative satisfaction score represented improvement from their presurgical status. This limits the ability of this study to establish causation with postoperative seizure control. Third, the retrospective design introduces several biases in our study, including recall bias. Although the ESSQ-19 questionnaire asks the patients to answer as they are currently feeling, or have felt in the past four weeks, the heterogeneity regarding the follow-up may have introduced this bias. Fourth, the patients in our study underwent two types of procedures: temporal lobe resection and corpus callosotomy, procedures with different therapeutic goals; the pooled satisfaction may obscure the differences between these two procedures. Lastly, the use of telephone interviews as the primary data collection method may elicit different responses than a self-administered tool, such as social desirability bias.

## Conclusions

This retrospective observational study reported patient satisfaction following epilepsy surgery at our tertiary care centre using a validated Epilepsy Surgery Satisfaction Questionnaire (ESSQ-19). In this study, high mean scores were observed in the surgical-complication (89.2±21.1) and seizure-control domains (85±27.6). Additionally, comparatively low scores were observed in the psychosocial domain (79.9±24.1). These findings suggest that although seizure control and surgical complications are important outcomes of epilepsy surgery, other quality of life indicators, such as psychosocial factors, also call for attention in the postoperative follow-up period.

The ESSQ-19 is a validated, comprehensive questionnaire that evaluates patient satisfaction following epilepsy surgery. However, given the limitations of this single-centre, retrospective study, these findings should be considered descriptive, limited to this cohort, and not generalized to other patient populations. Future large-scale, multicentric, prospective studies with larger sample sizes, preoperative baseline assessment, cultural validation of ESSQ-19 for the Indian population, and longer follow-up are needed to better understand the relationship between epilepsy surgery outcomes and patient satisfaction.

## References

[1] World Health Organization (2024). Epilepsy [Internet]. https://www.who.int/news-room/fact-sheets/detail/epilepsy.

[2] Kalilani L, Sun X, Pelgrims B, Noack-Rink M, Villanueva V (2018). The epidemiology of drug-resistant epilepsy: a systematic review and meta-analysis. Epilepsia.

[3] Kwan P, Brodie MJ (2000). Early identification of refractory epilepsy. N Engl J Med.

[4] Panayiotopoulos CP (2007). Old versus new antiepileptic drugs: the SANAD study. Lancet.

[5] Kwan P, Arzimanoglou A, Berg AT (2010). Definition of drug resistant epilepsy: consensus proposal by the ad hoc Task Force of the ILAE Commission on Therapeutic Strategies. Epilepsia.

[6] Bala A, Olejnik A, Kułak M (2024). Determinants of the quality of life in patients with drug-resistant temporal lobe epilepsy: a comparison of the results before and after surgery. Brain Sci.

[7] Yamamoto T (2020). Recent advancement of technologies and the transition to new concepts in epilepsy surgery. Neurol Med Chir (Tokyo).

[8] Sheng J, Liu S, Qin H, Li B, Zhang X (2018). Drug-resistant epilepsy and surgery. Curr Neuropharmacol.

[9] Taft C, Sager Magnusson E, Ekstedt G, Malmgren K (2014). Health-related quality of life, mood, and patient satisfaction after epilepsy surgery in Sweden--a prospective controlled observational study. Epilepsia.

[10] Wiebe S, Wahby S, Lawal OA (2020). Development and validation of the Epilepsy Surgery Satisfaction Questionnaire (ESSQ-19). Epilepsia.

[11] Revicki DA (2004). Patient assessment of treatment satisfaction: methods and practical issues. Gut.

[12] Burani M, Giovannini G, Pugnaghi M (2024). The Epilepsy Surgery Satisfaction Questionnaire (ESSQ-19): Italian language translation and validation. Neurol Sci.

[13] Yu Q, Ying YQ, Jiang SX, Xu ZY, Lan MJ, Guo Y (2023). Reliability and validation of the Chinese version of the epilepsy surgery satisfaction questionnaire. Epilepsy Behav.

[14] Fonseca E, Thomson A, Alonso M (2026). Spanish translation, adaptation, and validation of the Epilepsy Surgery Satisfaction Questionnaire-19. Epilepsia.

[15] Lin PT, Wiebe S, Chou CC (2022). Validation of the Taiwanese version of the Epilepsy Surgery Satisfaction Questionnaire (Tw-ESSQ-19). Epilepsy Behav.

[16] Rider F, Turchinets A, Zinchuk M (2025). The Epilepsy Surgery Satisfaction Questionnaire (ESSQ-19): linguistic adaptation and validation of the Russian version. Epilepsy Behav.

[17] Espinosa-Jovel C, Toledano R, Aledo-Serrano Á, García-Morales I, Gil-Nagel A (2018). Epidemiological profile of epilepsy in low income populations. Seizure.

[18] Rayner G, Wilson SJ (2012). Psychiatric care in epilepsy surgery: who needs it?: Psychiatric care in epilepsy surgery. Epilepsy Curr.

[19] Bakhtiar Y, Pratama Brilliantika S, Bunyamin J, Arifin MT, Hardian H, Bintoro AC, Muttaqin Z (2021). Postoperative evaluation of the quality of life, depression, and anxiety of temporal lobe epilepsy cohort: a single institute experience in Indonesia. Front Neurol.

[20] Hue CD, Couper RG, Antaya TC, Herrera M, Parra J, Burneo JG (2022). Depression and suicide after temporal lobe epilepsy surgery: a systematic review. Epilepsy Behav.

